# The Development of 7E Chromosome-Specific Molecular Markers for *Thinopyrum elongatum* Based on SLAF-seq Technology

**DOI:** 10.1371/journal.pone.0065122

**Published:** 2013-06-10

**Authors:** Shiqiang Chen, Zefeng Huang, Yi Dai, Shuwen Qin, Yingying Gao, Lulu Zhang, Yong Gao, Jianmin Chen

**Affiliations:** College of Bioscience and Biotechnology, Yangzhou University, Yangzhou, China; International Atomic Energy Agency, Austria

## Abstract

*Thinopyrum elongatum* is an important relative of wheat, it is favored by many researchers for the disease resistant genes that exist in its E genome. Some studies have showed that the 7E chromosome of *Th. elongatum* contains resistance genes related to *Fusarium* head blight and wheat rust. Therefore, developing 7E chromosome-specific molecular markers linked to resistance genes will provide an important tool for exploring and using the resistant genes of *Th. elongatum*. In addition, it would greatly contribute in the effort to cultivate disease-resistant wheat varieties. Featured in high throughput, high-accuracy and low-cost, SLAF-seq technology has been widely used in molecular breeding, system evolution, and germplasm resource detection. Based on SLAF-seq, 518 specific fragments on the 7E chromosome of *Th. elongatum* were successfully amplified. A total of 135 primers were designed according to 135 randomly selected fragments, and 89 specific molecular markers of *Th. elongatum* were developed, with efficiencies up to 65.9%. These markers were all detected in a variety of materials, and they are all proved to be specific and stable. These markers can be used not only for detecting the 7E chromosome of *Th. elongatum* but also for providing an important theoretical and practical basis for wheat breeding by marker-assisted selection (MAS). This paper reports the first application of SLAF-seq technology with a high success rate in developing specific molecular markers for *Th. elongatum,* providing a strong case for the application of this new technology.

## Introduction

*Thinopyrum elongatum* (syn. *Lophopyrum elongatum* or *Agropyron elongatum*) is an important wild relative of wheat, belonging to the tribe *Triticeae* and genus *Elytrigia.* It contains three species based on the ploydity: diploid (2*n* = 2*X* = 14, EE, syn. E^e^E^e^), tetraploid (2*n* = 4*X* = 28, E^e^E^e^E^b^E^b^) and decaploid (2*n* = 10*X* = 70, E^e^E^e^E^b^E^b^E^x^E^x^StStStSt). The E genome of the diploid is the basic genome of *Th. elongatum* [1], [2]. In addition, hexaploid *Th. elongatum* (2*n* = 6*X* = 42) was alsoreported [3]. *Th. elongatum* has the same ancestor with common wheat, which exhibits relatively small genetic differentiation between its E and its A, B, and D genomes [1], [2]. *Th. elongatum* mainly grows in temperate and cold zones, and is a perennial herb with many superior characteristics, such as long spikes, multi-flowers, high grain protein content, strong adaptability and reproductive ability. As it has some useful genes for adverse conditions such as disease, cold, drought and salinity, it is regarded as an important potential gene donor for improving biotic and abiotic stress tolerance in wheat [2], [4]–[12]. Chinese and American scientists have developed several wheat varieties using common wheat and *Th. elongatum*, such as Xiaoyan 6 [13], [14]. This shows that *Th. elongatum* can play an important role in the genetic improvement of wheat. After Chinese Spring-*Th. elongatum* addition and substitution lines were bred successfully, the beneficial characteristics of *Th. elongatum*, such as stress resistance and good quality, were widely studied at the chromosome level [10]–[12], [15], [16].

*Fusarium* head blight (FHB) and wheat rust are prevalent wheat diseases and can cause a great reduction in wheat production. Although there are some resistant resources in common wheat germplasm [17], [18], they still cannot control the occurrence of FHB and wheat rust, and they cannot meet the needs of wheat resistance breeding. The study of *Th. elongatum* has been particularly interesting to researchers world-wide. Studies have shown that the 7E chromosome of *Th. elongatum* contains some resistance genes [7], [8], [10]–[12], [19], such as the anti-FHB gene *FhbLoP* [10] and the anti-rust gene *Lr19* [10], [20]–[22]. Therefore, fully developing and utilizing the resistance genes in the 7E chromosome of *Th. elongatum* will greatly enrich wheat resistance resources.

Marker-assisted selection (MAS) is a method to select good linkage genes or breeding multi-gene varieties based on molecular markers [23]. It is necessary and important to develop molecular markers linked to the genes beneficial for plant breeding by MAS. With many excellent genes on the *Th. elongatum* chromosomes, developing a large number of related, specific molecular markers will improve the chances of obtaining markers tightly linked to anti-disease genes. The markers also can improve the accuracy of anti-disease identification and further accelerate the use of *Th. elongatum*. In fact, several *Th. elongatum* chromosome-specific molecular markers have been developed by RAPD [24], [25], SSR [1], [14], [26], RFLP [27], AFLP [20], [28], STS [28], SCAR [22], [25], [29], CAPS [30], RGAP [31], TRAP [14], and SSH [32]. With the high genomic sequence homology between *Th. elongatum* and common wheat and the weaknesses of current technologies listed above due to high cost, long cycle, and low success rate in molecular marker development, it is difficult to obtain the large amount of markers needed to meet the requirement for breeding anti-disease varieties by MAS.

The SLAF-seq (Specific Length Amplified Fragment Sequencing) was developed based on high-throughput sequencing technology. It allows researchers to design the experimental system through bioinformatics and screen for fragments of a specific length from the constructed SLAF-seq library. The massive sequences were then obtained and analyzed using SLAF_Poly.pl. (Biomarker, Beijing, China). After a sequence comparison using BLAT [33], a large number of specific fragments are selected for specific molecular markers development. SLAF-seq technology has several obvious advantages, such as high throughput, high accuracy, low cost and short cycle, which enable its sequencing results to be directly used for molecular markers development. This technology has been reported for haplotype mapping, genetic mapping, linkage mapping, and polymorphism mapping. It can also provide an important basis for molecular breeding, system evolution and germplasm resource identification. In this paper, SLAF-seq technology was first used to obtain *Th. elongatum* 7E chromosome-specific fragments and to successfully develop many 7E chromosome-specific molecular markers. The success of developing chromosome- specific molecular markers by SLAF-seq technology provides a strong technical support for its future application.

## Materials and Methods

### Materials

The genetic stocks employed in the current study are listed in Table 1, including Chinese Spring (CS), diploid *Thinopyrum elongatum* (*Th. elongatum*, 2*n* = 2*X*), their addition lines (DA lines), ditelo addition lines (DA7ES, DA7EL), substitution lines (DS lines), other wheat varieties, polyploid *Th. elongatum*, and cross offsprings. The materials were supplied by Dr. Goeger Fedak (Eastern cereal and oilseed research center, Canada), academician Shunhe Cheng (Lixiahe region agricultural scientific research institute, China).

**Table 1:** The experimental materials used in this study.

Name of the Materials	Abbreviation of the Materials
Diploid *Thinopyrum elongatum*	*Th. elongatum* (2*n* = 2*X*)
Chinese Spring	CS
Chinese Spring-*Thinopyrum elongatum* disomic addition lines	DA1E, DA2E, DA3E, DA4E, DA5E, DA6E, DA7E
Chinese Spring-*Thinopyrum elongatum* telodisomic addition lines	DA7ES,DA7EL
Chinese Spring-*Thinopyrum elongatum* disomic substitution lines	DS1E(1A), DS1E(1B), DS1E(1D), DS2E(2A), DS2E(2B), DS2E(2D), DS3E(3A), DS3E(3B),
	DS3E(3D), DS4E(4A), DS4E(4B), DS4E(4D),
	DS5E(5B), DS5E(5D), DS6E(6A), DS6E(6D), DS7E(7A), DS7E(7B), DS7E(7D)
Langdon	LD
Yangmai 10, Yangmai 14, Yangmai 16, Yangmai 18, Yangmai 158	Y10, Y14, Y16, Y18, Y158
Ningmai 13	N13
Annong 8455	An8455
Sumai 3	Su3
Langdon-*Th. elongatum* amphidiploid	8801
Tetraploid *Thinopyrum elongatum*	*Th. elongatum* (2*n* = 4*X*)
Decaploid *Thinopyrum elongatum*	*Th. elongatum* (2*n* = 10*X*) (PI179162/PI204383)
F_1_ and F_2_ of Yangmai 16×DS7E(7A)	YD-F_1_, YD-F_2_
F_1_ and F_2_ of DS7E(7A)×Yangmai 16	DY-F_1_, DY-F_2_

### SLAF-seq Technology Scheme Design

Based on the GC content, repeat sequences and gene characters, the wheat BAC sequences were analyzed using SLAF_Predict (Biomarker, Beijing, China). The plan for marker development was designed by defining the enzyme digestion scheme, gel cutting ranges and sequencing quantity, which were used to verify the density and homogeneity of the marker being developed and ensure the likelihood of successfully preparing the expected target.

### Genomic DNA Extraction

The SDS method [34] was used to extract genomic DNA from young leaves of the genetic stocks. DNA quality and concentration were measured by 0.8% agarose gel electrophoresis, and adjustments were made for a final DNA concentration of 100 ng µL^−1^.

### Genomic DNA Digestion

Genomic DNA (500 ng) from CS, *Th. elongatum* (2*n* = 2*X*) and DA7E were incubated at 37°C with 0.6U MseI (New England Biolabs, Hitchin, Herts, UK), T_4_ DNA ligase (NEB), ATP (NEB) and MseI adapters. Restriction-ligation reactions were heat-inactivated at 65°C and then digested in an additional reaction with the restriction enzymes HaeIII and BfaI at 37°C.

### PCR Reaction and Fragment Amplification

A PCR reaction was performed containing the diluted restriction-ligation samples, dNTP, Taq DNA polymerase (NEB) and MseI-primer containing barcode. The PCR products were purified by E.Z.N.A.® Cycle Pure Kit (Omega) and pooled.

### Fragment Selection, Extraction and Amplification

The pooled sample was incubated at 37°C with MseI, T_4_ DNA ligase, ATP and Solexa adapters. The samples were purified using a Quick Spin column (Qiagen) and then separated on a 2% agarose gel to isolate the fragments between 300 to 500 bp using a Gel Extraction Kit (Qiagen). These fragments were used in a PCR amplification with Phusion Master Mix (NEB) and Solexa amplification primer mix. Phusion PCR settings followed the Illumina sample preparation guide. Samples were gel-purified, and products with appropriate sizes (300 to 500 bp) were excised and diluted for sequencing by Illumina GAIIx (Illumina, San Diego, CA, USA).

### Sequencing and Sequence Analysis

The cluster density was optimized to ensure that the SLAFs corresponding with the set requirements, and the PCR amplified products were sequenced using an Illumina GAIIx (Illumina, CA, USA). The SLAFs were identified and filtered to ensure that the original sequencing data were effectively obtained. They were clustered based on similarity using BLAT [33], and their sequences were obtained through focused recognition and correction techniques.

### Sequence Comparison and *Thinopyrum elongatum* 7E Chromosome-specific Fragment Acquisition

The fragments of DA7E and *Th. elongatum* (2*n* = 2*X*) were selected by a specificity comparison. The sequences with good quality from *Th. elongatum* (2*n* = 2*X*) and DA7E were first compared with the CS sequences acquired by SLAF-seq, and they were then compared with the sequences on www.ncbi.nlm.nih.gov and www.cerealsdb.uk.net. Finally, the specific sequences of DA7E and *Th. elongatum* (2*n* = 2*X*) were compared and the 7E chromosome-specific sequences of *Th. elongatum* were obtained.

### 7E Chromosome-specific Molecular Markers of *Thinopyrum elongatum* Development and Stability Detection

Based on these sequences, PCR primers were designed for the amplification of DA7E and CS. The amplified products were electrophoresed in 0.8% agarose gel, and the markers presented in DA7E but absent in CS were identified as the 7E chromosome-specific molecular markers. Then, the stabilities of these markers were detected in DA lines, DA7ES, DA7EL, DS lines, CS, *Th. elongatum* (2*n* = 2*X*), LD, Y10, Y14, Y16, Y18, Y158, N13, An8455, Su3, 8801, *Th. elongatum* (2*n* = 4*X*), *Th. elongatum* (2*n* = 10*X*), YD-F_1_, YD-F_2_, DY-F_1_ and DY-F_2,_ respectively. The PCR system contained 1 µL of genomic DNA (100 ng µL^−1^), 2.5 µL of loading buffer (10×), 2 µL of dNTP (2.5 mM), 1 µL of primer 1 (10 µM) and primer 2 (10 µM), 0.3 µL of Taq (5 U µL^−1^) and 18.2 µL of double-distilled water. The PCR procedure was as follows: 94°C for 5 min; followed by 35 cycles of 94°C for 45 s, appropriate anneal temperature (45–60°C) for 1 min, and 72°C for 1.5 min; then 72°C for 10 min.

## Results and Analysis

### Acquisition of Specific Sequences from the 7E Chromosome of *Thinopyrum elongatum*

Using the SLAF-seq technology, 70,152, 49,848 and 59,141 effective SLAFs were acquired for CS, *Th. elongatum* (2*n* = 2*X*) and DA7E, respectively. The sequencing depth was more than 9×. The result was optimal and fulfilled the expected requirements. After comparing the CS sequences acquired by SLAF-seq and the sequences in www.ncbi.nlm.nih.gov or www.cerealsdb.uk.net, 20,170 *Th. elongatum* (2*n* = 2*X*) and 4,984 DA7E sequences whose homology with CS and other wheat species was less than 50% were selected as the specific sequences for *Th. elongatum* (2*n* = 2*X*) or DA7E. From those specific ones, 518 DA7E sequences with homologies higher than 80% of *Th. elongatum* (2*n* = 2*X*) were obtained. These DA7E sequences were identified as the 7E chromosome-specific sequences of *Th. elongatum*.

### Primer Design and Marker Development for 7E Chromosome of *Thinopyrum elongatum*

**Figure 1:**
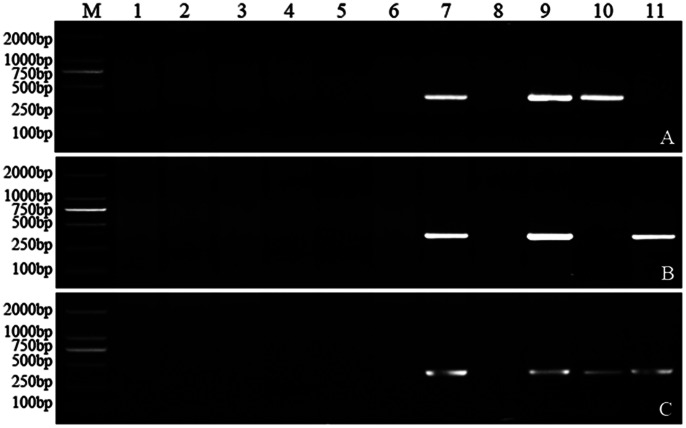
The PCR amplification of M7E_No.1 (A), M7E_No.2 (B) and M7E_No.9 (C) in CS- *Thelongatum* disomic addition and 7E telodisomic addition lines. M: Marker (DL2000); 1–7: DA1E-DA7E; 8: CS; 9: *Th. elongatum* (2*n* = 2*X*); 10: DA7ES; 11: DA7EL.

Based on 135 sequences randomly selected from the specific sequences of the 7E chromosome, 135 pairs of primers were designed for developing specific molecular markers (Table 2). PCR products were amplified from DA lines (DA1E-7E), CS, *Th. elongatum* (2*n* = 2*X*), DA7ES and DA7EL, respectively. A total of 89 of *Th. elongatum* specific molecular markers were successfully developed (Table 2), with the success rate up to 65.9%. These markers included 61 *Th. elongatum* 7E chromosome specific markers, 14 genome markers and 14 chromosome markers which also appeared on several other chromosomes including 7E. The 61 specific molecular markers of the 7E chromosome included 35 only appearing on the short arm of the 7E chromosome (Fig. 1A), 24 on the long arm (Fig. 1B), and 2 on both arms (Fig. 1C). The 14 genome markers included 1 marker that only appeared on the short arm, 1 on the long arm and 12 on both arms of the 7E chromosome. The 14 other markers included 8 that only appeared on the short arm, 4 on the long arm and 2 on both arms of the 7E chromosome. The success rate of developing the 7E chromosome-specific molecular markers was as high as 45.2%.

**Table 2:** The 7E chromosome-specific molecular markers and PCR primers of *Thinopyrum elongatum.*

Specific Markers	SpecificPrimers	Sequences of the Special Primers (5′–3′)			AmplifiedChromosomes	Original Fragments
		Forward	Reverse			
M7E_No.1	P7E_No.1	ATCAATCCCTCCACAAAGTC		GCCTTTTTTTTCTAATGTGC	7E,7ES	SLAF119658
M7E_No.2	P7E_No.2	AGCAAATAAAACGACAGT		GGTTTTCACCAATTACAG	7E,7EL	SLAF31945
M7E_No.3	P7E_No.3	GGAAAATGGAATCAAGGAGG		GAACTCGTATCCCTTGCCTT	1E-7E,7ES,7EL	SLAF2140
M7E_No.4	P7E_No.4	TTTTTCTGGTACTTACTAAC		TGTTCTGTGAGATGAATGTT	7E,7ES	SLAF5720
M7E_No.5	P7E_No.5	AGTCAAGGCAAATTATGGT		ACTGTCTAATGTCTCGTAAT	7E,7EL	SLAF48385
M7E_No.6	P7E_No.6	GGTGTGTAGAAAAACATAAC		CTTTGAATACACCCTACTTA	1E,7E,7ES	SLAF45083
M7E_No.7	P7E_No.7	ATTTTGTCCCTATGCT		ATGCCTTCCTGATTTA	7E,7ES	SLAF49074
M7E_No.8	P7E_No.8	ACCATTCCCCAAAACT		ATTGTCAATTATTTTTTAGC	1E-4E,7E,7ES,7EL	SLAF20928
M7E_No.9	P7E_No.9	AGTGAACTGAATTCTCTGCT		CTCTTTTGAAGCATTACACA	7E,7ES,7EL	SLAF50207
M7E_No.10	P7E_No.10	TTTCGCTGCTGAAGAATCTA		GCAAAACCATCATACAATCG	7E,7ES,7EL	SLAF140297
M7E_No.11	P7E_No.11	TAGAATAGCTTTTAGGAATA		GTGAGCATTCTTAGCATTAC	7E,7ES	SLAF19795
M7E_No.12	P7E_No.12	AAACCCAAATGAAAGT		CAAACTTATAAGTAGAGACA	7E,7ES	SLAF25934
M7E_No.13	P7E_No.13	CACTTGTGCATATTCGAGAG		CCATTTTCCAATATATACAA	7E,7ES	SLAF1998
M7E_No.14	P7E_No.14	GAAATGGCAAGTACCTAA		CAAGTAGAAGTTCAGCAA	7E,7ES	SLAF47229
M7E_No.15	P7E_No.15	CCAAGAAAAGTCAACCGT		GAGCAACCAAATTCAATAGA	7E,7EL	SLAF44157
M7E_No.16	P7E_No.16	CCTCGAAATCAATCAATCCT		CCATAAAAAGGCAAAAATCC	7E,7ES	SLAF21225
M7E_No.17	P7E_No.17	CAGCACCACTGTTTACTTAG		ACACCTGTAAGGCTGTAATA	1E-7E,7EL	SLAF11089
M7E_No.18	P7E_No.18	ATGTTGTTGTTTTTTGGTGT		TGGAAACTTGTATGAAATGG	7E,7EL	SLAF237685
M7E_No.19	P7E_No.19	TTTCCATTGGGGTAGT		CGGGGTGATTACTTTC	7E,7ES	SLAF29032
M7E_No.20	P7E_No.20	CTTTCCATAGTAGGTCCAGT		CGAACTTTGTTTGAAATATG	7E,7ES	SLAF49101
M7E_No.21	P7E_No.21	GCACAAAAAGAGCAGAATAT		AAGCTTATTACAGGACCATG	7E,7ES	SLAF22240
M7E_No.22	P7E_No.22	AGTGAATCTGAGATGCATAT		ATTTTGTTTTTACCATTTTT	7E,7ES	SLAF27230
M7E_No.23	P7E_No.23	GCTCTTGAGCGTCTACAGTG		TTCGTATGGTTTTTTCTGGC	7E,7ES	SLAF65372
M7E_No.24	P7E_No.24	CAAAATGAAAAGATAAAACT		AGATTCAAAATTTTAGTTAT	7E,7ES	SLAF91582
M7E_No.25	P7E_No.25	CAGCTCCATCGAAACTCT		GGACGACCTGCTAATACA	5E,7E,7EL	SLAF46357
M7E_No.26	P7E_No.26	TGTGTCGTAGAGATGTGTTG		GGGTGATAGACATATGCAAT	7E,7EL	SLAF46632
M7E_No.27	P7E_No.27	GTCGTGGATATGTCATTGTA		TTGTATGGATGCTTTGGT	1E,2E,4E,5E,7E,7ES	SLAF258417
M7E_No.28	P7E_No.28	ATTCTGATGTGTATTGAGCC		AACGTGTCCACTAACAACTT	7E,7ES	SLAF31380
M7E_No.29	P7E_No.29	CTACTGCTTTAGGGTGTTGA		CCAAGAATAGCACAAACAAC	7E,7ES	SLAF22799
M7E_No.30	P7E_No.30	AAGTTCAAGTTTGCAGGTAC		AGCATTAGTATTTGAGAAGC	4E,5E,7E,7ES	SLAF238
M7E_No.31	P7E_No.31	CTACCCTTACCACCTCG		CCACTGGATGCTGTTTAT	7E,7ES	SLAF14034
M7E_No.32	P7E_No.32	CTGAGCTGCGTCGGTA		CCAGAAATTGCTAAAATCTT	1E-7E,7ES,7EL	SLAF35615
M7E_No.33	P7E_No.33	TGTTTAGTAGAGGGTTCATT		GTGTGGGTAATATTTTTGTA	1E-7E,7ES	SLAF44977
M7E_No.34	P7E_No.34	AAAATCAGCGGTGCCT		ACCTGTAGATTGAAATGCCT	7E,7EL	SLAF49963
M7E_No.35	P7E_No.35	GACCAATGGAAAGAAAATGT		CAACACTCTTGTCTTCCTTT	7E,7EL	SLAF42598
M7E_No.36	P7E_No.36	TGTTTCTTAGTTGTTTTGTT		GCCTTGACCACCATAC	1E-7E,7ES,7EL	SLAF12623
M7E_No.37	P7E_No.37	GGTAAGCTTGAAATACATGA		TCCAAGTGATATTGTAGTCG	7E,7ES	SLAF32494
M7E_No.38	P7E_No.38	GTGGAATTGGACTTTTTTTG		AGATTTCCTGTTATCCCAAG	1E-7E,7ES,7EL	SLAF231806
M7E_No.39	P7E_No.39	TTTATAAGTTGATGAGGGGG		AAGGCTTTACCGAAAATCAT	7E,7EL	SLAF216573
M7E_No.40	P7E_No.40	CTCGTCCTCGTCCTCCTTGT		AGCATAACTTGCCAATCCCC	7E,7ES	SLAF5918
M7E_No.41	P7E_No.41	AAAGTGCTTCATCCCAAAT		AGGATGATATGAATGCTTTT	7E,7EL	SLAF14218
M7E_No.42	P7E_No.42	TTAGCATATGCTTTTTAGGC		GCAAATCAGTTCAGTGAACC	1E,3E-5E,7E,7ES,7EL	SLAF12080
M7E_No.43	P7E_No.43	GCCCAGTGTAGTTCGCTCGT		TTCTCAGGCGAGGAAGTGGA	7E,7ES	SLAF39853
M7E_No.44	P7E_No.44	ACAGATGCCTAAAAGC		CACAAAATCTTGGGTC	7E,7EL	SLAF7994
M7E_No.45	P7E_No.45	TTGTTTGTTGGACTTGAATG		GCACAAAATAGTGAGAAGGC	7E,7ES	SLAF8447
M7E_No.46	P7E_No.46	GTCTAACTTGTTGTGTGTGC		CACTCAGGAACTAAATTTGC	7E,7EL	SLAF12583
M7E_No.47	P7E_No.47	ATGTTGTACTCCATTCAGAT		GAGATACAAAAATTTGAGTG	1E,5E,7E,7ES	SLAF24261
M7E_No.48	P7E_No.48	CATGGGTGATGAAAAGAAGA		GCCAACTATGTGGTTTCAAG	7E,7ES	SLAF236334
M7E_No.49	P7E_No.49	ATACTTGAGGTGATTTCGGT		GGTGCAAAGTTTTTACAATG	7E,7ES	SLAF236809
M7E_No.50	P7E_No.50	TAAAGTGGAGGTAAAATGAC		AAAGATTCGAAAAATTAGTT	7E,7EL	SLAF200585
M7E_No.51	P7E_No.51	TACACAGAAGGAAAGCATTA		CATCAGAAATTTTCTTTTGA	7E,7EL	SLAF1699
M7E_No.52	P7E_No.52	ACAAGTCCATTCATTACAAC		TACTACTTTTGTGACAGCAG	7E,7ES	SLAF43910
M7E_No.53	P7E_No.53	GTCAAGAGTTGGCTTTATTC		ATTTGCTAATTCTCGTCATA	7E,7ES	SLAF6445
M7E_No.54	P7E_No.54	CATGCGACCTACAATAAATT		GTAATTTTTTGTCATGTGCC	7E,7EL	SLAF251157
M7E_No.55	P7E_No.55	ATTATTTACGTTTCTTGAGC		CTTCCCCACTCTTTGACT	1E,2E,5E-7E,7EL	SLAF140771
M7E_No.56	P7E_No.56	TTACACTAACCCATGGTGTT		GCAGAGAATGAAGCAAAATC	7E,7ES	SLAF12105
M7E_No.57	P7E_No.57	CTTTTATGTATTTGAGAGCA		CGCAACTCCAATATGA	7E,7ES	SLAF22820
M7E_No.58	P7E_No.58	CAAATCTGTTGAACTGTCTT		TGCGATACAAGTATAAAATG	5E,7E,7ES,7EL	SLAF15563
M7E_No.59	P7E_No.59	TTGCTACAAATATTGAGTCA		GTACTTGTGCATCCCTTC	7E,7ES	SLAF130591
M7E_No.60	P7E_No.60	TTTTCCAGCTTCCTAATT		TTGACTGCTTCATTCTTC	5E,7E,7ES,7EL	SLAF160814
M7E_No.61	P7E_No.61	TAAGTTGATAGATGTGCTG		TTGAATTGTAGCTAAAGTAA	1E-7E,7ES,7EL	SLAF15482
M7E_No.62	P7E_No.62	CCAAGATGGTATGACACTAT		AGTACTCGGATGATTTTCTC	7E,7ES	SLAF29906
M7E_No.63	P7E_No.63	ACAAGCAGAATCGGAACG		GCACATCCAATTGTCACACT	1E-7E,7ES,7EL	SLAF105525
M7E_No.64	P7E_No.64	ATTTTATGACCAAGGACT		ACACACACTTCTACTTTC	4E,5E,6E,7E,7ES	SLAF137880
M7E_No.65	P7E_No.65	CACACACTTCTACTTTCG		GGGTTGGTTCCATCACAT	7E,7ES	SLAF137880
M7E_No.66	P7E_No.66	GGGTTTACCTCCGCATCG		GCAAATTATTATCAGCCACCAA	2E-7E,7ES	SLAF3970
M7E_No.67	P7E_No.67	ATTTTGTCAGTGGAATGGAT		AATAAATCAAATCCTGCTCA	4E,5E,7E,7ES	SLAF251334
M7E_No.68	P7E_No.68	CAATGGTACATATCACACT		ATGCACGATTCTACAGT	7E,7ES	SLAF774
M7E_No.69	P7E_No.69	TTTCTGTAAGCCGATGC		AAGAACTACCTGGTGAAATAC	1E-7E,7ES,7EL	SLAF45682
M7E_No.70	P7E_No.70	AATGGAGCCCAAGGAG		CCATCCAACGGAAGTG	1E-7E,7ES,7EL	SLAF16926
M7E_No.71	P7E_No.71	GTCTTGCCTGTCCTCG		ATTTTCAAAGTTCTCACAAG	7E,7EL	SLAF252555
M7E_No.72	P7E_No.72	GGACTTGGACTCTATCTTC		GACCCAACAATTTCGA	7E,7EL	SLAF45552
M7E_No.73	P7E_No.73	ACTCATACCAATCCCGTCTA		TTGTTATTTTCGCACTATGG	1E-7E,7ES,7EL	SLAF40006
M7E_No.74	P7E_No.74	CGTGCCTGTGGTTATGT		TTGCCTTCAGTCATTTCA	7E,7EL	SLAF9221
M7E_No.75	P7E_No.75	TTCAAAGGAACATTTACAAG		CTACCCGGTCCTTCTC	1E-7E,7ES,7EL	SLAF362764
M7E_No.76	P7E_No.76	AGCATAGGGACCACTTC		TTACTGATGGATTGGCA	1E-7E,7ES,7EL	SLAF23848-
M7E_No.77	P7E_No.77	TGTTGTAGTTTCGTCCCT		TGGTGGATGAGGAAGAC	7E,7ES	SLAF4571
M7E_No.78	P7E_No.78	AATTACTATGTGCATCGG		TGTAATCAAAATATCAGTCG	7E,7EL	SLAF129639
M7E_No.79	P7E_No.79	GTAGTATCTCGCCGATGTCGT		TCTGGCGTGATTATTGTGGC	1E-7E,7ES,7EL	SLAF9285
M7E_No.80	P7E_No.80	GCTTGGAGGAGTTGAT		TTCTTCTATGTGTTTTATTG	7E,7EL	SLAF32358
M7E_No.81	P7E_No.81	ACACAAAGGTGAGTGAAAAC		GAGTAGCAAAAATCTCAACA	7E,7EL	SLAF72555
M7E_No.82	P7E_No.82	AGTATTGTGCCAGTATTC		ATCAAGAGGGTATAACTG	7E,7ES	SLAF34164
M7E_No.83	P7E_No.83	AGACTATCTTATCAACCATT		CAACTACACGCTAAACC	7E,7ES	SLAF38680
M7E_No.84	P7E_No.84	CGAAGGGTCTTTGATT		GCAAACATCTGACAAGG	7E,7E	SLAF2228
M7E_No.85	P7E_No.85	CATGTTTACGTCCTAATTCT		TCAAACTGCTTGCTCTG	7E,7EL	SLAF3153
M7E_No.86	P7E_No.86	CACCATTGCAAGTTTGA		AAGCCCACCTCTATTGA	7E,7EL	SLAF69129
M7E_No.87	P7E_No.87	ACAAACCAATGGAAAGG		CGGAGCAACTACAGACG	2E,6E,7E,7ES	SLAF35412
M7E_No.88	P7E_No.88	ATGTTCTTTCTTTCGGTT		GCTTACTCAACAGAAAAAAC	7E,7ES	SLAF1128
M7E_No.89	P7E_No.89	TGCAATGTCCTTGATAGA		GCTCTGTAAAGGTAAAATCT	7E,7EL	SLAF240072

A list of the names of the specific markers is shown, where M7E_No.1 stands for the first (No.1) molecular marker (M) of the *Thinopyrum elongatum* 7E-chromosome (7E). A list of the name of the specific primers is shown, where P7E_No.1 stands for the first pair (No.1) of primers (P) of the *Thinopyrum elongatum* 7E-chromosome (7E). Additionally, the name of the original fragments is listed, where SLAF119658 stands for specific (S) length (L) amplified (A) fragment (F), and its number is 119658.

### Repeatablity, Stability and Specificity of the 7E Chromosome- specific Molecular Markers of *Thinopyrum elongatum*

**Figure 2:**
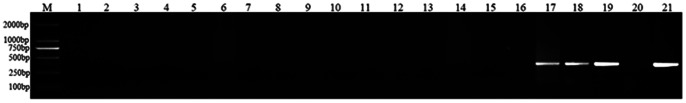
The stability of M7E_No.2 in CS- *Th. elongatum* disomic substitution lines. M: Marker (DL2000); 1: DS1E (1A); 2: DS1E (1B); 3: DS1E (1D); 4: DS2E (2A); 5: DS2E (2B); 6: DS2E(2D); 7: DS3E(3A); 8: DS3E(3B); 9: DS3E(3D); 10: DS4E(4A); 11: DS4E(4B); 12: DS4E(4D); 13: DS5E(5B); 14: DS5E(5D); 15: DS6E(6A); 16: DS6E(6D); 17: DS7E(7A); 18: DS7E(7B); 19: DS7E(7D); 20: CS; 21: *Th. elongatum* (2*n* = 2*X*).

**Figure 3:**
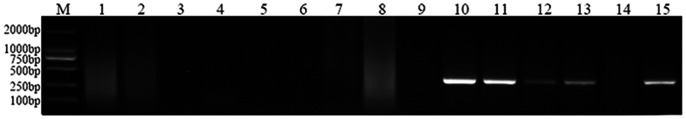
The stability of M7E_No.2 in other wheat, amphidiploid and polyploid *Th. Elongatum*. M: Marker (DL2000); 1: LD; 2: Y10; 3: Y14; 4: Y16; 5: Y18; 6: Y158; 7: N13; 8: An 8455; 9: Su 3; 10∶8801; 11: *Th. elongatum* (2*n* = 4*X*); 12: *Th. elongatum* (2*n* = 10*X*, PI179162); 13: *Th. elongatum* (2*n* = 10*X*, PI204383); 14: CS; 15: *Th. elongatum* (2*n* = 2*X*).

**Figure 4:**
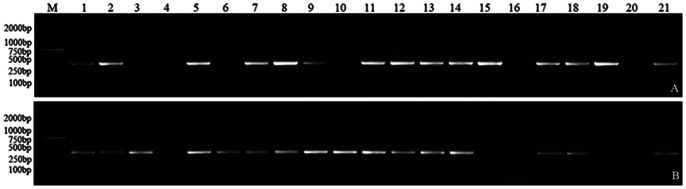
The stability of M7E_No.2 in F_1_ and F_2_ of orthogonal (A) and reciprocal (B) cross offspring of Y16 and DS7E (7A). M:Marker (DL2000); 1: YD-F_1_ (A) or DY-F_1_ (B); 2–19: YD-F_2_ (A) or DY-F_2_ (B); 20: Y16; 21: DS7E(7A).

To test for the repeatability, stability and specificity, the molecular markers were amplified using all materials listed in Table 1, including DA lines, DA7ES, DS7EL, DS lines, CS, *Th. elongatum*, other wheat varieties, polyploid *Th. elongatum*, and cross offsprings. The results showed that the markers developed by the SLAF-seq technology were repeatable, stable and specific. For example, M7E_No.2 appeared consistently in DA7E, DA7EL, *Th. elongatum* (2*n* = 2*X*) (Fig. 1B), DS7E(7A), DS7E(7B), DS7E(7D) (Fig. 2), 8801, *Th. elongatum* (2*n* = 4*X*), *Th. elongatum* (2*n* = 10*X*) (Fig. 3), YD-F_1_, DY-F_1_, parts of YD-F_2_ and DY-F_2_ (Fig. 4), while it did not appear in the materials lacking the 7E chromosome. This indicated that M7E_No.2 is a repeatable, stable and specific molecular marker of *Th. elongatum* 7E chromosome.

### Analysis of the 7E Chromosome-specific Molecular Markers of *Thinopyrum elongatum*

PCR products of ten markers randomly selected from the 89 specific molecular markers of *Th. elongatum* were re-sequenced and compared with common wheat sequences. As expected, the lengths of the specific molecular markers of *Th. elongatum* developed by SLAF-seq were between 300 bp to 500 bp, and they hadlittle sequence homology with common wheat. To confirm these findings, M7E_No.2. was re-sequenced and compared with wheat common sequences in www.ncbi.nlm.nih.gov or www.cerealsdb.uk.net. It showed that the 339 bp M7E_No.2 marker (Table 3) had low sequence homology with CS or other common wheat varieties.

**Table 3:** The DNA sequences and length of the *Thinopyrum elongatum* 7E chromosome-specific molecular marker M7E_No.2.

Name	Sequences(5′-3′)	Length
M7E_No.2	AGCAAATAAAACGACAGTGCAGCTCGTGGTTAGTATGAAAATTTACTTTAGTATACTACTATCCGCATCTAATGCATGTATGGATGCACCAAAATTTGTACTAATAAAGGAGCATTATCATATTTGTTTAGCAAGCGAACCGTGGTACTTATTGCAGCAGAACACTTCTGAATAAATTCAATGCGGGAGAGAGGTGTTACCTTCTTAGCATTCAGGTAGCTGTCCTTGGGTAGCTCGGTAAAGGTATTTTTCAAAGGAGTTCTCGACCCGGTGCTCCATGGTGCAGTATCCAGTGACGATGCAATTAGCAGACAGCCCCGGCTGTAATT GGTGAAAACC	339 bp

## Discussion

### The Feasibility and Advantages of SLAF-seq Technology in Chromosome-specific Molecular Marker Development

SLAF-seq technology is highly automated because it was developed using bioinformatics for high-throughput sequencing technology applications. It can generate large amounts of sequence information and handle any whole genome density distributions. In this study, 518 specific fragments of the 7E chromosome of *Th. elongatum* were obtained by the SLAF-seq technology. Based on 135 randomly selected fragments, 89 specific molecular markers including 61 7E-chromosome specific molecular markers were developed. SLAF-seq technology was capable of developing *Th. elongatum* specific markers with high success rate and low cost. On the other hand, the success rate of developing *Th. elongatum* genome- or chromosome-specific molecular markers by conventional methods were quite low [24], [26], [28], [32]. For example, 94 *Th. elongatum* specific fragments were obtained using 26 pair of RAPD primers [24] with only 3 1E or 3E chromosome-specific molecular markers obtained. 108 *Th. elongatum* specific fragments were obtained using 40 SSR primers [26] with only 1 genome-specific molecular markers obtained. 28 *Th. elongatum* specific fragments were obtained using 5 pair of AFLP primers [28] with only 4 chromosome-specific molecular markers obtained. In addition, 65 *Th. elongatum* specific fragments were obtained using SSH, but only 1 chromosome-specific molecular marker was developed [32]. The SLAF-seq technique cost 1/8 of that of AFLP while the efficiency was 27 times (www.biomarker.com.cn). Therefore, compared to RAPD [24], AFLP [28] or SSH [32], the SLAF-seq technology is much better in developing plant chromosome-specific molecular markers with higher success rate, specificity, stability, and lower cost.

### Repeatability, Stability and Specificity of the *Thinopyrum elongatum* 7E Chromosome-specific Molecular Markers Developed by SLAF-seq

M7E_No.2, one 7E chromosome-specific molecular marker, uniquely appeared in all the materials containing the 7E chromosome but not in others (Fig. 1B, 2, 3 and 4). This suggested that M7E_No.2 was reliable and the fact M7E_No.2 stably appeared not only in the diploid *Th. elongatum* but also in the polyploid *Th. elongatum* proved that the E genome of the diploid *Th. elongatum* was the basic genome of the polyploid *Th. elongatum* (Fig. 3). M7E_No.2 was detected in some progenies of YD-F_2_ and DY-F_2_, and its segregation of positive and negative was nearly 3∶1, strictly consistent with Mendel’s law (Fig. 4).

All the specific molecular markers of *Th. elongatum* were also detected, and the results, especially those of the 60 7E-chromosome specific molecular markers, were the same as that of M7E_No.2. This finding showed that the specific molecular markers of *Th. elongatum* developed by the SLAF-seq technology were all repeatable, stable and specific. The result of the 14 genome markers and the other 14 chromosome markers also appearing in the materials having some E chromosomes confirmed that all the E chromosomes of *Th. elongatum* had high DNA sequence homology with each other which might be caused by chromosomal rearrangement [27].

### The Application Value of the 7E Chromosome-specific Molecular Markers of *Thinopyrum elongatum*

After DA lines and DS lines were crossed successfully, the positive characteristics of *Th. elongatum* were widely studied at the chromosome level [10]–[12], [15], [16]. Dvorák et al. found that different chromosomes of *Th. elongatum* had different effects, whereas the 7E chromosome affected the number of days to heading, maturity and seed yield, decreased the plant height, and increased the seed weight [15], [16]. Many studies also showed that there were anti-FHB genes [7], [8], [10]–[12], [19] and anti-rust genes [10], [20], [21], such as *FhbLoP* or *Lr19,* located on the 7E chromosome of *Th. elongatum*. If the resistance genes are fully explored and used, they would greatly enrich the resistance germplasm resources for wheat.

The 7E chromosome-specific molecular markers of *Th. elongatum* developed in this study are dominant markers, which provides a good basis for their subsequent applications. Based on molecular markers, *FhbLoP* has been mapped to the very distal region of the long arm of 7E chromosome within a 3.71 cM interval flanked by *Xcfa2240* and *Xswes19*, which accounts for 30.46% of the phenotypic variance. *Lr19* has been bracketed by *Xwmc273* and *XBE404744*, with a map distance of 1.54 and 1.43 cM from either side, respectively [10]. The closely linked markers to anti-disease genes will be helpful for marker-assisted introgression of the genes of interest, such as anti-FHB genes, into elite cultivars of the common wheat. The development of a genetic map will accelerate the map-based cloning of these genes. Hybridizing or back-crossing between DS lines and cultivated wheat, or using *Ph* gene mutation, small fragments containing resistance genes of *Th. elongatum* E genome will translate into wheat which can be performed rapidly and accurately to obtain the resistance offspring by MAS [14], [35]. It was reported that radiating the hybrid offspring between DS lines and cultivated resulted in the chromosome fragments to break and reclose, allowing the generation of *Th. elongatum* translocation lines. Using the MAS, these translocation lines can be used to breed anti-desease wheat varieties [14], [36].

Developing a large number of *Th. elongatum* 7E chromosome-specific molecular markers is very valuable, not only for the identification of *Th. elongatum* 7E chromosomes but also for the acceleration of the exploration and usage of the useful genes of *Th. elongatum* with high agronomical or anti-disease value, such as *FhbLoP* and *Lr19*. This finding further enriches the resistance resources for wheat and provides a basis for anti-disease or anti-stress wheat breeding.
